# Natural killer cell activated by attenuated newcastle disease virus (NDV) as anti-cancer therapy

**DOI:** 10.3389/fmolb.2026.1721060

**Published:** 2026-03-19

**Authors:** Noor N. Al-Hayani, Marwa I. Salman, Ahmed Majeed Al-Shammari

**Affiliations:** 1 College of Medicine, University of Anbar, Ramadi, Iraq; 2 Department of Biotechnology, College of Science, University of Baghdad, Baghdad, Iraq; 3 Experimental Therapy, Iraqi Center for Cancer and Medical Genetics Research, Mustansiriyah University, Baghdad, Iraq

**Keywords:** natural killer (NK) cells, Newcastle disease virus (NDV), oncolytic immunovirotherapy, synergistic antitumor activity, translational cancer immunotherapy

## Abstract

**Background:**

Natural Killer (NK) cells play a central role in innate immunity by targeting virally infected and malignant cells without prior sensitization. However, their activity is often suppressed within the tumor microenvironment. Oncolytic viruses such as Newcastle Disease Virus (NDV) not only selectively replicate in tumor cells but also stimulate immune responses, particularly NK cell activation. Combining attenuated NDV with NK cell therapy may therefore enhance anti-tumor efficacy.

**Aim:**

This study aimed to investigate the synergistic anti-cancer potential of NK cells activated by attenuated NDV against breast cancer cell lines AMJ13 and MCF-7, with emphasis on cytotoxicity, adhesion, and immunophenotypic changes.

**Methods:**

NK cells were isolated from peripheral blood using separation media and 8 µm mesh filtration, followed by expansion in culture with interleukin-15 (IL-15). Immunofluorescence assays were performed to characterize these NK cells by immunophenotyping through detection of CD3, CD16, Cd56 and CD57 expressions. Co-cytotoxicity was evaluated by WST assay in AMJ13 and MCF-7 cells exposed to NK cells, NDV, or the combination. NK adhesion to tumor cells was assessed by light microscope, scanning electron microscopy (SEM) and immunofluorescence by CD56 detection. Statistical analysis was conducted using GraphPad Prism, and combination effects were analyzed by CompuSyn software.

**Results:**

NK cells expanded effectively in IL-15–supplemented culture and displayed enhanced cytotoxicity when combined with NDV, leading to significantly reduced viability in both AMJ13 and MCF-7 cells compared with single treatments (p < 0.05). light and SEM analyses demonstrated NK cell adhesion, morphological alterations, and surface disruption of tumor cells. Immunofluorescence confirmed increased expression of NK marker (CD56) combination in treated groups, supporting functional enhance attachemtn of NK cells.

**Conclusion:**

Attenuated NDV significantly augments NK cell-mediated cytotoxicity and adhesion against breast cancer cells. This combinatorial approach offers a promising immunotherapeutic strategy, highlighting the potential of integrating oncolytic virotherapy with NK cell-based therapy for cancer treatment.

## Introduction

Natural killer (NK) cells are innate lymphocytes capable of recognizing and eliminating malignant or virally infected cells without prior antigen sensitization. This occur through the integration of activating and inhibitory receptor signals that tune responsiveness to cellular stress and loss of self-MHC I (Vivier et al., 2011). Their ability to mediate antibody-dependent and natural cytotoxicity makes them attractive candidates for adoptive cell therapy (Nigro et al., 2019). Over the past decade, NK cell based therapies have entered early clinical success, including the first U.S. FDA-cleared off-the-shelf product (Fate Therapeutics FT500) and multiple allogeneic NK products in phase I-II trials for solid and hematologic malignancies (Lamers-Kok et al., 2022). Cytokine-activated NKs and memory-like NKs have demonstrated safety, minimal graft-versus-host risk, and measurable antitumor activity (Liu et al., 2020; Marin et al., 2024). Despite this intrinsic capability, many solid tumors develop immune-evasion programs down-modulating activating ligands, inducing immunosuppressive cytokines, and remodeling the microenvironment to evade NK cytotoxicity and persistence (Wu et al., 2020).

Oncolytic viruses (OVs) are now an established therapeutic class combining direct tumor lysis with immune activation. The U.S. FDA approval of talimogene laherparepvec (T-VEC) for melanoma validated this concept clinically (Andtbacka et al., 2015). Moreover, OVs have emerged as immunotherapeutics that combine direct oncolysis and cancer cell killing with remodeling and changing the tumor microenvironment (TME) toward inflammation and immune cell recruitment (Kaufman et al., 2015). There are numerous viral platforms; adenovirus, vaccinia, vesicular stomatitis virus (VSV), measles virus, and Newcastle disease virus (NDV) are under clinical evaluation (Alwithenani et al., 2025; Russell et al., 2012). One of the most studied OVs, Newcastle disease virus (NDV), which is avian paramyxovirus non-pathogenic to humans, displays selective replication in and cytotoxicity against malignant cells, while triggering type I interferon signaling and danger responses (Zamarin and Palese, 2012). NDV infection can also increase the immunogenicity of tumor cells and promote innate immune activation; for example, NDV hemagglutinin-neuraminidase (HN) inserted on infected tumor cells surface can engage NKp44/NKp46, and NDV can upregulate adhesion and interferon-stimulated programs that favor effector–target interactions (Jarahian et al., 2009). Both naturally occurring and attenuated NDV strains induce intrinsic and extrinsic tumor apoptosis, autophagy, glycolysis inhibition and immunogenic cell death (Al-Shammari et al., 2012; Elankumaran et al., 2006; Jiang et al., 2014; Obaid et al., 2021).

These properties suggest a rational synergy between NDV and NK cells: NDV primes tumor targets and inflames the TME, while NK cells perform potent contact-dependent cytotoxicity and immune amplification. Prior studies have demonstrated that NDV infection sensitizes tumor cells to NK lysis by upregulating ligands such as NKG2D and TRAIL receptors (Wang et al., 2022). and that NK–virus interaction can yield synergistic tumor suppression in preclinical models (Zamarin et al., 2014). In this study, we investigated the synergistic antitumor activity of attenuated NDV and primary NK cells *in vitro* against breast carcinoma cells. We integrated multiple complementary approaches to provide mechanistic evidence that NDV primes breast cancer cells for enhanced NK recognition and cytolysis, supporting the development of NK-NDV immunovirotherapy as a translationally relevant anticancer strategy. Although NDV-mediated NK activation has been previously described, a systematic quantitative evaluation of NDV-NK synergy using formal combination index modeling, dose-reduction analysis, and structural visualization of effector target interactions in breast cancer models has not been performed.

## Materials and methods

### Cell lines and culture conditions

The human breast adenocarcinoma cell line MCF-7 (ATCC HTB-22) and the AMJ13 Iraqi breast cancer cell line (Al-Shammari et al., 2015) were maintained in MEM and RPMI-1640 medium (Capricorn-Scientific, Germany) supplemented with 10% fetal bovine serum (FBS), 1% penicillin-streptomycin, and incubated at 37 °C. Cells were sub-cultured at 70%–80% confluence and routinely tested negative for *Mycoplasma*.

### Newcastle disease virus (NDV)

An attenuated NDV AMHA1 strain was propagated in 9-day-old embryonated chicken eggs, harvested from allantoic fluid, clarified by low-speed centrifugation, and further purified by ultracentrifugation over glucose density gradient, aliquoted, and stored at −80 °C. Viral titer was determined by the hemagglutination (HA) assay and expressed as hemagglutination units (HAU). Multiplicity of infection (MOI 5, 10, 20) was calculated based on viable tumor cell counts at seeding. Mock controls received virus-free allantoic fluid.

The Newcastle disease virus strain used in this study (NDV-AMHA1) is an attenuated strain previously isolated and characterized by our group (Al-Shammari et al., 2014a; Al-Shammari et al., 2014b). The strain has been evaluated in multiple *in vitro* and *in vivo* cancer models, demonstrating tumor-selective replication and induction of apoptosis with limited cytotoxicity in normal cells (Al-Shammari and Salman, 2024; Hamad et al., 2024; Al-Sh et al., 2023; Kadhim et al., 2023).

Attenuation characteristics of NDV-AMHA1 are supported by its biological behavior consistent with non-velogenic NDV strains. Complete viral genome sequencing has been performed using next-generation sequencing platforms to confirm strain identity and genomic stability. Due to ongoing intellectual property considerations related to translational development, the full genome sequence has not yet been publicly deposited but is available upon reasonable academic requests.

### Isolation and expansion of natural killer (NK) cells

Peripheral blood mononuclear cells (PBMCs) were obtained from healthy donors under ethical approval and informed consent by ICCMGR, Mustansiriyah University and College of Medicine, University of Anbar scientific committees’ number 626 on 12 December 2024. NK cells were isolated using density gradient lymphocyte separation media (Capricorn-Scientific, Germany), followed by filtration through an 8 μm mesh of SPLInsert™ Hanging (SPL, South Korea) to enrich NK fractions through natural migration ability for NK cells. Isolated NK cells cultured in RPMI-1640 medium supplemented with 10% fetal bovine serum (FBS) and antibiotics (penicillin-streptomycin). To promote expansion, cells were stimulated with recombinant human IL-15 (20 Ng/mL). Cultures maintained at 37 °C in a humidified 5% CO_2_ incubator, with media replacement every 2–3 days. Cell proliferation monitored microscopically.

### Immunophenotyping for isolated NK cells

For immunophenotyping, NK cells (4 × 10^4^ cells/well) were seeded onto poly-L-lysine–coated 8-well chamber slides (SPL, South Korea) and allowed to adhere overnight at 37 °C. Cells were fixed in 4% paraformaldehyde (PFA) for 15 min, washed twice with phosphate-buffered saline (PBS), and permeabilized with 0.1% Triton X-100 for 10 min. Non-specific binding was blocked using 5% bovine serum albumin (BSA) in PBS for 30 min at room temperature. Samples were incubated for 60 min with panel of NK specific primary antibodies (Elabscience, China) diluted 1:100 in blocking buffer. The following antibodies were used; FITC-conjugated Mouse anti-human CD3; CD56; CD16 and CD57. After three PBS washes for 5 min, followed by final washes and mounting with mounting medium (Elabscience, China). Fluorescence imaging was performed using a Micros (Austria) upright fluorescence microscope equipped with a digital camera and appropriate FITC filter sets. Exposure settings were kept constant across all samples.

### Cytotoxicity and synergy analysis

Both AMJ13 and MCF-7 breast cancer cell lines were seeded in 96-well plates at 5 × 10^3^ cells/well (100 μL; n ≥ 3 technical replicates per condition) and allowed to adhere overnight. NK cells were isolated and maintained in IL-15 supplemented medium. Effector inputs were 500, 1,000, or 2,000 NK cells/well (final volume 200 µL), corresponding to approximate E:T ratios of 1:10, 1:5, and 1:2.5, respectively. For treatments, four conditions were tested in parallel: untreated control, NDV alone (MOI 5, 10, or 20), NK alone (500, 1000, 2000 cells/well), and NDV + NK combinations at the same dose levels. NDV was added directly to the culture medium; for combination modality; NDV was added first for 2 h, and NK cells were added after these 2 h. Plates were incubated 72 h at 37 °C/5% CO_2_ unless otherwise stated. Cell viability was quantified using WST reagent (Elabscience, China). Briefly, 10 μL of CCK-8 was added per well and incubated 2 h at 37 °C, then absorbance was read at 450 nm. Background-subtracted values were normalized to the means of untreated controls (set to 100% viability). % Killing was calculated as

$$\%\text{ Killing}=\left[1\;–\;\left(\text{Absorbance sample }/\text{ Absorbance control}\right)\right]\times 100$$

Here, Absorbance sample refers to the absorbance measured in wells containing treated cells (e.g., with NK cells, NDV, or both), while Absorbance control refers to the absorbance from untreated control wells. This formula enables quantification of the reduction in cell viability as a percentage, relative to the control group.

Synergy modeling (Chou-Talalay) (Chou, 2010). For each cell line, single-agent dose-effect data (NDV MOIs 5–20; NK 500–2,000/well) and the corresponding combination data were exported to CompuSyn. Median-effect parameters (Dm, m, r) were obtained for NDV and NK, and fraction affected (Fa) values were computed as Fa = 1 – viability. Combination Index (CI) values were generated for predefined dose pairs; CI < 1 denotes synergy, CI = 1 additivity, and CI > 1 antagonism. Dose-Reduction Index (DRI) values for each agent were also derived. Outputs included dose-effect curves, median-effect plots, Fa-CI plots, DRI plots, and normalized isobolograms, which were used to construct Figures 2, 3. For WST endpoints, data are presented as mean ± SD from ≥3 independent fields/wells per condition and ≥3 independent experiments where available.

### Acridine orange/propidium iodide (AO/PI) dual fluorescence apoptosis assay

Apoptotic and late-stage cell death following NDV and NK cell treatment were evaluated using acridine orange/propidium iodide (AO/PI) dual fluorescence staining. AMJ13 and MCF-7 cells were seeded in 8-well chamber slides at a density of 1 × 10^4^ cells per well and allowed to adhere overnight under standard culture conditions (37 °C, 5% CO_2_). Experimental groups included untreated control, NDV alone (MOI 20), NK cells alone (2000 cells/well), and combined NDV (MOI 20) + NK cells (2000 cells/well). After 72 h of incubation, culture media were gently aspirated, and wells were washed once with phosphate-buffered saline (PBS) to remove non-adherent cells while preserving effector-target contacts. AO and PI were freshly prepared in PBS at final concentrations of 10 μg/mL each, and cells were incubated with the staining solution for 5 min at room temperature in the dark. Excess dye was removed by gentle PBS washing, and slides were immediately analyzed using a fluorescence microscope (Micros, Austria) equipped with FITC and Texas Red filter sets under identical exposure settings for all conditions. Cells were classified based on fluorescence signal and nuclear morphology as follows: viable cells exhibited uniform green nuclear fluorescence (AO-positive, PI-negative); early apoptotic cells displayed bright green nuclei with chromatin condensation or fragmentation; late apoptotic cells demonstrated orange to red fluorescence with condensed or fragmented nuclei; and necrotic cells showed uniform red fluorescence without characteristic apoptotic morphology. For quantitative analysis, three independent microscopic fields per condition derived from separate wells (n = 3 biological replicates) were randomly selected and imaged. Fluorescence intensity and cell counts were quantified using ImageJ software (NIH, United States), and the percentages of AO-positive viable cells and PI-positive late apoptotic/necrotic cells were calculated. Data are presented as mean ± standard deviation (SD). Statistical comparisons were performed using one-way ANOVA followed by Tukey’s multiple comparisons test, with p < 0.05 considered statistically significant.

### NK adhesion assay (cytology and SEM)

#### Morphological assessment by light microscopy

AMJ13 and MCF-7 cells were seeded in 8-well chamber slides (SPL, South Korea) and grown to about 70% confluence. Four conditions were tested: untreated control, NDV only (MOI 20; per standard procedure and replaced with fresh medium), NK only (2,000 cells/well), and NDV + NK (MOI 20 + 2,000 NK cells/well). Co-incubations proceeded for 24 h unless otherwise stated. Monolayers were first examined directly in the chamber slides under brightfield/phase contrast to document NK adherence and gross cytopathic changes. Imaging was performed on a Micros (Austria) upright microscope equipped with the digital camera, using 10×-4×0 objectives. Exposure/illumination settings were kept constant within each experiment. Qualitative endpoints included NK adhesion to tumor cells, and tumor cytopathic changes (rounding, shrinkage, membrane collapse, detachment, debris). Multiple non-overlapping fields per well and ≥3 wells per condition were captured to ensure representation.

#### Immunofluorescence staining for CD56 and quantitative analysis

To evaluate NK cell adherence to tumor cells, immunofluorescence staining for CD56 was performed on AMJ13 and MCF-7 cultures grown in 8-well chamber slides (SPL, South Korea). Experimental conditions included untreated control, NDV infection alone, NK cells alone, and combined NDV + NK co-treatment.

Cells were washed gently with phosphate-buffered saline (PBS) and fixed with 4% paraformaldehyde (PFA; Santacruz Biotechnology, United States) for 15 min at room temperature. Following fixation, cells were permeabilized with 0.1% Triton X-100 in PBS for 10 min and blocked with 5% bovine serum albumin (BSA; Thermo Fisher Scientific, United States) for 1 h to minimize nonspecific binding. Chambers were incubated for 1 h at room temperature with FITC-conjugated mouse anti-human CD56 monoclonal antibody (Elabscience, China) diluted 1:100 in blocking buffer. After final PBS washes, chamber walls were removed, and slides were mounted with mounting medium (Elabscience, China). Fluorescence images were acquired using an inverted fluorescence microscope (Micros, Austria) with a FITC filter set and digital camera. Exposure times and acquisition parameters were kept identical across all groups. For quantitative analysis, CD56^+^ NK cells were identified from the FITC channel using ImageJ (NIH, United States). Images underwent background correction (morphological opening), Gaussian smoothing, adaptive thresholding, and binarization. Small objects (<40 pixels) were excluded. Each discrete CD56^+^ fluorescent object was counted as a single adherent NK cell. Counts were normalized to field area (based on the 200 µm scale bar) and expressed as cells per 200 × 200 µm field equivalent. Data was compiled from multiple independent fields (n ≥ 3 per condition) and summarized as mean ± SD. Statistical analyses were performed in GraphPad Prism v9.0 using one-way ANOVA with Tukey’s post-hoc test. p values <0.05 were considered statistically significant.

#### Immunofluorescent detection of NDV hemagglutinin-neuraminidase (HN)

To confirm productive NDV infection in tumor cells, hemagglutinin-neuraminidase (HN) protein expression was assessed by immunofluorescence. AMJ13 and MCF-7 cells were seeded in 8-well chamber slides and infected with attenuated NDV-AMHA1 at MOI 20. After 24 h, cells were washed with PBS and fixed in 4% paraformaldehyde for 15 min at room temperature. Following permeabilization with 0.1% Triton X-100 for 10 min, non-specific binding was blocked using 5% bovine serum albumin (BSA) in PBS for 1 h.

Cells were incubated overnight at 4 °C with primary antibody against NDV HN protein (NDV HN Antibody (HN14f): sc-53562, Santa Cruz Biotechnology, Inc, Texas, United States). After washing, samples were incubated with FITC-conjugated secondary antibody (Immunol Fluorescence Staining Kit (Anti-Mouse IgG-FITC) (E-IR-R324), Elabscience, China) for 1 h at room temperature in the dark. Slides were mounted using anti-fade mounting medium.

Fluorescence images were acquired using a Micros (Austria) fluorescence microscope under identical exposure settings for all conditions. Mean fluorescence intensity (MFI) per microscopic field was quantified using ImageJ software following background subtraction. Three independent microscopic fields derived from separate wells were analyzed per condition. Statistical analysis was performed using unpaired Student’s t-test. Data are presented as mean ± SD.

#### Scanning electron microscopy (SEM)

For ultrastructural analysis of NK-tumor interactions, AMJ13 and MCF-7 cells were seeded at 20000 cells/mL and cultured on sterile 12-mm round glass coverslips placed in 24-well plates until reaching 70% confluence. Experimental conditions included untreated controls, NDV infection alone (moi of 20), NK cells alone (2000 cells), and combined NDV + NK co-treatment (moi of 20 + 2000 cells). Following incubation, cultures were washed with phosphate-buffered saline (PBS) and fixed in 2.5% glutaraldehyde for 2 h at room temperature. Samples were then rinsed in buffer. After that, specimens were dehydrated in a graded ethanol series (30%, 50%, 70%, 90%, 100%; 10 min each) and air-dried briefly. Dried coverslips were mounted on aluminum SEM stubs using double-sided conductive carbon tape and further dried and sputter-coated with a 5 nm gold layer using a Luxor® Gold Plasma Coater (Luxor Tech, Germany), to increase surface conductivity and minimize charging. Samples were examined using a Phenom ProX G5 Desktop SEM (Thermofisher, Netherlands) operated at 10–15 kV in high-vacuum mode. Images were captured at multiple magnifications (500×–12,500×) to evaluate overall tumor cell morphology, NK-tumor cells interaction, and evidence of cytotoxic antitumor activity. Morphological features of interest included NK adherence, immune synapse formation, tumor membrane blebbing, surface perforations, and detachment. Representative images were selected from replicate experiments for figure presentation.

### Statistical analysis

All experiments were performed in triplicates. Data expressed as mean ± standard deviation (SD). Statistical comparisons were made using one-way ANOVA followed by Tukey’s post-hoc test. A p-value <0.05 was considered significant. Combination studies (NDV + NK cells) were analyzed for synergy, additivity, or antagonism using CompuSyn software. All quantitative data are presented as mean ± standard deviation (SD) from three independent biological replicates (n = 3). Statistical significance among multiple treatment groups was determined using one-way ANOVA followed by Tukey’s *post hoc* multiple comparison test (GraphPad Prism v6). Exact p-values were calculated for all pairwise comparisons. A p-value <0.05 was considered statistically significant.

## Results

### Immunophenotypic characterization of isolated NK cells

**FIGURE 1:**
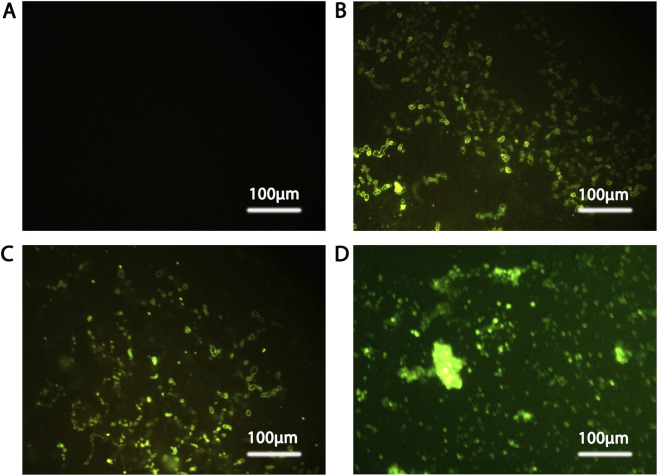
Immunophenotypic characterization of NK cells isolated by natural migration and used in functional assays. Primary NK cells were enriched from peripheral blood mononuclear cells (PBMCs) using a natural migration-based selection approach and maintained in IL-15 supplemented medium prior to downstream assays. Immunofluorescence staining was performed to validate lineage identity and maturation status. **(A)** CD3 staining shows absence of detectable signal, excluding T-lymphocyte contamination. **(B)** CD56 expression identifies NK lineage cells within the enriched population. **(C)** CD16 positivity demonstrates the presence of cytotoxic NK subsets associated with antibody-dependent and direct target cell killing. **(D)** CD57 staining identifies a subset of NK cells exhibiting features consistent with functional maturation. Images were acquired under identical exposure settings for all markers. Scale bar = 100 μm. Collectively, these findings indicate that the enriched effector population consisted predominantly of CD3^−^CD56^+^ NK cells with detectable CD16 and CD57 expression. Precise discrimination between CD56^bright^ and CD56^dim^ subsets requires flow cytometric analysis and was therefore not inferred from fluorescence intensity in these images.

Freshly isolated NK cells obtained by natural migration demonstrated >95% viability and were successfully maintained in IL-15 supplemented culture, yielding sufficient effector cells for downstream cytotoxicity and immunophenotyping assays. Immunofluorescence analysis confirmed the NK identity of the expanded effector population. CD3 staining was consistently negative (Figure 1A), excluding T-cell contamination. In contrast, robust CD56 expression was observed (Figure 1B), confirming enrichment of the NK lineage. CD16 staining was detected in a substantial proportion of CD56^+^ cells (Figure 1C), supporting the presence of cytotoxic NK subsets capable of antibody-dependent and direct target cell killing. In addition, CD57 positivity was observed in a subset of CD56^+^ cells (Figure 1D), indicating partial maturation within the expanded NK population.

Because discrimination between CD56^bright^ and CD56^dim^ subsets requires flow cytometric resolution, subset stratification was not inferred based on fluorescence intensity alone. However, the co-detection of CD16 and CD57 within the CD56^+^ population supports the presence of a functionally competent cytotoxic NK compartment. Together, these findings demonstrate that the effector cells used in subsequent experiments were predominantly CD3^−^CD56^+^ NK cells enriched for cytotoxic and partially mature phenotypes. Because immunofluorescence imaging was performed in separate staining panels rather than multiplexed single-cell co-expression analysis, precise quantification of CD16^+^ and CD57^+^ subset frequencies within the CD56^+^ population was not determined.

### NDV-NK Co-treatment potentiates breast cancer cells killing

**FIGURE 2:**
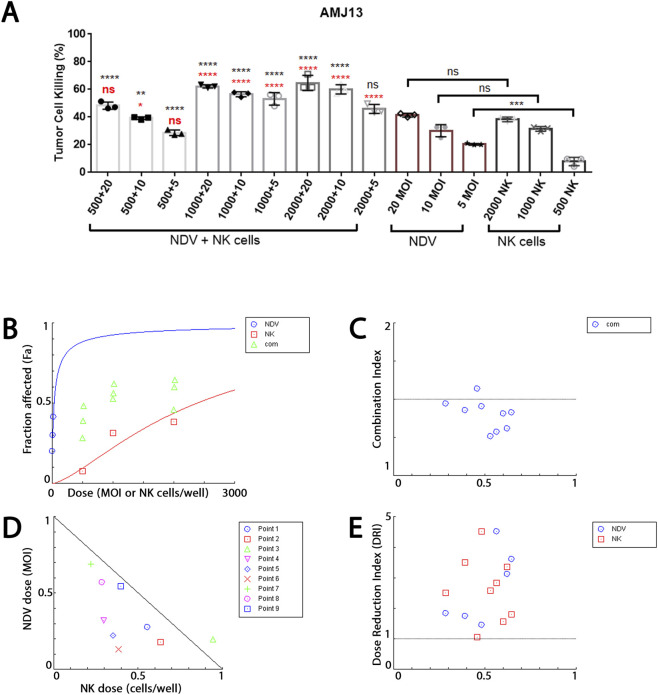
Synergistic cytotoxicity of attenuated NDV and primary NK cells against AMJ13 breast carcinoma cells. **(A)** WST-based cytotoxicity assay demonstrating tumor cell killing following treatment with NDV alone (MOI 5, 10, 20), NK cells alone (500, 1000, 2000 cells/well), or combined NDV + NK regimens. Single-agent treatments induced moderate cytotoxicity (≤41% for NDV at MOI 20; ≤38% for NK at 2000 cells/well), whereas combination therapy significantly enhanced tumor cell killing, reaching approximately 65%–70% at MOI 20 + 2000 NK cells. Red asterisks indicate comparison versus corresponding NDV-only treatment; black asterisks indicate comparison versus corresponding NK-only treatment. **(B)** Median-effect dose-response curves generated using CompuSyn software demonstrate dose-dependent cytotoxicity for NDV and NK monotherapies, with an upward-shifted combination curve consistent with enhanced efficacy. **(C)** Combination Index (CI) versus fractional effect (Fa) plot showing CI < 1 across the majority of dose combinations, confirming synergistic interaction between NDV and NK cells. Strong synergy was observed at intermediate combinations (e.g., MOI 5–10 + 1000 NK cells; CI ≈ 0.52–0.57), with only one regimen (MOI 5 + 2000 NK cells) showing slight antagonism (CI = 1.14). **(D)** Dose-Reduction Index (DRI) analysis indicating substantial dose-sparing in combination therapy. At Fa = 0.6, NDV dose requirements decreased approximately 5.5-fold and NK cell input decreased approximately 1.6-fold relative to monotherapy conditions. **(E)** Normalized isobologram analysis demonstrating that nearly all experimental combination points lie below the theoretical additivity line, further confirming synergistic interaction. Data represent mean ± SD from three independent biological experiments (n = 3). Statistical significance was determined by one-way ANOVA followed by Tukey’s multiple comparisons test. A p-value <0.05 was considered statistically significant. Synergy analysis was performed using the Chou-Talalay median-effect method implemented in CompuSyn software.

**FIGURE 3:**
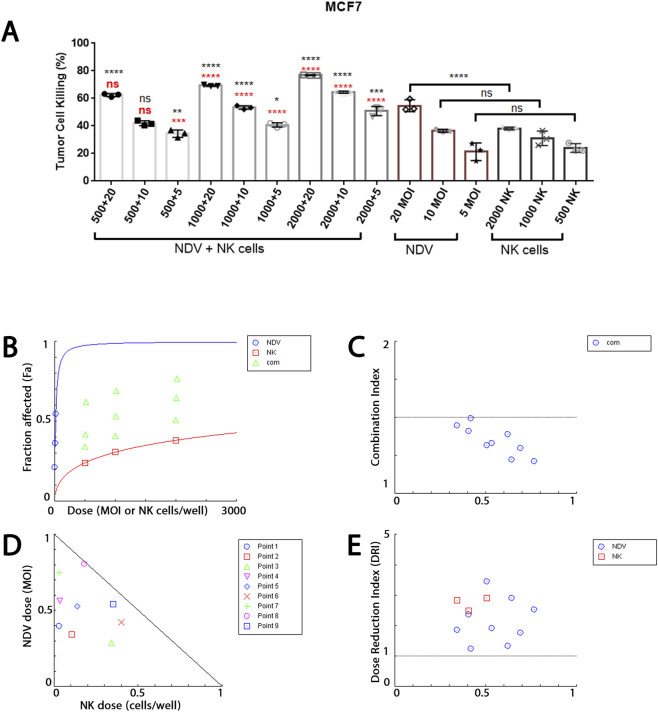
Synergistic cytotoxicity of attenuated NDV and primary NK cells against MCF-7 breast carcinoma cells. **(A)** WST-based cytotoxicity assay demonstrating tumor cell killing following treatment with NDV alone (MOI 5, 10, 20), NK cells alone (500, 1000, 2000 cells/well), or combined NDV + NK regimens. Single-agent treatments induced moderate cytotoxicity (≤55% for NDV at MOI 20; ≤38% for NK at 2000 cells/well), whereas combination therapy significantly enhanced tumor cell killing, reaching approximately 75%–78% at MOI 20 + 2000 NK cells. Red asterisks indicate comparison versus corresponding NDV-only treatment; black asterisks indicate comparison versus corresponding NK-only treatment. **(B)** Median-effect dose response curves generated using CompuSyn software demonstrate dose-dependent cytotoxicity for NDV and NK monotherapies, with an upward-shifted combination curve consistent with enhanced efficacy. **(C)** Combination Index (CI) versus fractional effect (Fa) plot showing CI < 1 across the majority of dose combinations, confirming synergistic interaction between NDV and NK cells. Strong synergy was observed at intermediate and high fractional effects, with CI values ranging approximately 0.43–0.82 across most combinations. **(D)** Dose-Reduction Index (DRI) analysis indicating substantial dose-sparing in combination therapy. At higher fractional effects (Fa ≈0.75), NK dose requirements were markedly reduced, demonstrating enhanced therapeutic efficiency relative to monotherapy conditions. **(E)** Normalized isobologram analysis demonstrating that nearly all experimental combination points lie below the theoretical additivity line, further confirming synergistic interaction. Data represent mean ± SD from three independent biological experiments (n = 3). Statistical significance was determined by one-way ANOVA followed by Tukey’s multiple comparisons test. A p-value <0.05 was considered statistically significant. Synergy analysis was performed using the Chou-Talalay median-effect method implemented in CompuSyn software.

To establish baseline antitumor activity, we first assessed the cytotoxic effects of NDV and NK cells, alone or in combination, using the WST viability assay in AMJ13 and MCF-7 breast carcinoma cells. As single agents, both NDV and NK cells produce moderate, dose-dependent cytotoxicity. In AMJ13 cells (Figure 2A), NDV reduced viability by 41% at MOI 20, while NK cells at 2000 per well achieved 38% inhibition. Similarly, in MCF-7 cells (Figure 3A), NDV at MOI 20 reduced viability by 39%, and NK cells at 2000 per well achieved 35% inhibition. Lower doses of either agent (MOI 5–10 or ≤1000 NK cells) induced only partial killing (≤25–30%).

In contrast, combined NDV-NK treatment markedly amplified cytotoxicity in both cell lines. In AMJ13 cultures, MOI 20 + 2000 NK cells achieved 68% killing, significantly exceeding the effect of either agent alone (p < 0.0001). In MCF-7 cells, the same regimen produced 77% inhibition, representing the strongest response observed across both models. Importantly, intermediate regimens (e.g., MOI 10 + 1000 NK cells) yielded 55%–56% killing in both lines, underscoring that supportive effects were not restricted to the highest doses.

Statistical comparisons confirmed that most combination regimens were significantly more effective than monotherapies, with only a few low-dose combinations failing to reach statistical significance. Collectively, these findings demonstrate that NDV and NK cells each exert modest cytotoxicity against breast cancer cells when applied individually, but their co-administration consistently produces enhanced killing, establishing a functional basis for synergy analysis.

### Chou-Talalay median-effect and combination index (CI) analysis confirms synergy

To rigorously quantify the interaction between NDV and NK cells, WST viability data were analyzed using the Chou-Talalay median-effect method implemented in CompuSyn. Median-effect plots demonstrated excellent conformity to the mass-action law, with linear regression correlation coefficients (r) exceeding 0.92 for all datasets. For AMJ13 cells (Figure 2B), the median-effect dose (Dm) values were 31.85 MOI for NDV and 2373.6 cells/well for NK cells, with slope (m) values of 0.74 and 1.44, respectively (r = 0.9999 and 0.93). For MCF-7 cells (Figure 3B), NDV exhibited a lower Dm of 16.78 MOI with an m of 1.08 (r = 0.99999), while NK cells required 5446.8 cells/well to achieve 50% effect (m = 0.48, r = 0.9994). These parameters confirm predictable, dose-dependent killing by each agent individually. Combination index values across a wide range of fractional effects (Fa) demonstrated robust synergy (CI < 1) for most NDV–NK dose pairs in both cell lines. Representative conditions are summarized in Table 1. AMJ13 cells (Figure 2C).: CI values as low as 0.52 were observed at MOI 5 + 1000 NK cells (Fa = 0.531), and 0.57 at MOI 10 + 1000 NK cells (Fa = 0.564). Only one regimen, MOI 5 + 2000 NK cells, showed slight antagonism (CI = 1.14). MCF-7 cells (Figure 3C).: Synergy was even more pronounced, with CI values of 0.43 (MOI 20 + 2000 NK, Fa = 0.767) and 0.45 (MOI 10 + 2000 NK, Fa = 0.645). Across all nine tested combinations, CI values ranged from 0.43 to 0.99, confirming consistent synergy.

**TABLE 1:** Combination Index (CI) values for NDV-NK co-treatment in AMJ13 and MCF-7 breast cancer cells.

NDV (MOI)	NK cells (per well)	Fraction affected (Fa)	CI value	Interaction
A. AMJ13 cells				
20	2000	0.646	0.8318	Moderate synergy
10	2000	0.600	0.8165	Moderate synergy
5	2000	0.459	1.1409	Slight antagonism
20	1000	0.622	0.6171	Strong synergy
10	1000	0.564	0.5735	Strong synergy
5	1000	0.531	0.5191	Strong synergy
20	500	0.483	0.9096	Additive/mild synergy
10	500	0.392	0.8559	Moderate synergy
5	500	0.286	0.9423	Additive
B. MCF-7 cells				
20	2000	0.767	0.4273	Strong synergy
10	2000	0.645	0.4491	Strong synergy
5	2000	0.508	0.6327	Strong synergy
20	1000	0.692	0.5984	Strong synergy
10	1000	0.534	0.6637	Strong synergy
5	1000	0.407	0.8240	Moderate synergy
20	500	0.624	0.7785	Moderate synergy
10	500	0.419	0.9872	Additive
5	500	0.344	0.8931	Moderate synergy

### Dose-Reduction Index (DRI) demonstrates therapeutic gain

Synergy translated into substantial dose-sparing for both effectors. AMJ13 (Figure 2D): At Fa = 0.6, the NDV dose was reduced to about 5.5-fold (DRI = 5.53) and NK requirement about 1.6-fold. At Fa = 0.75, NDV DRI was 4.5 and NK DRI 2.8. MCF-7 (Figure 3D): At Fa = 0.767, NDV DRI was 2.5, while NK DRI reached 32.6, underscoring a profound sparing effect on NK cell input. Even at lower Fa (0.407), NDV and NK doses were reduced to 2.37- and 2.49-fold, respectively.

### Isobologram validation

Normalized Isobologram plots confirmed the CI analyses, with nearly all data points falling below the line of additivity, visually indicating synergy. Collectively, these findings quantitatively establish that NDV and NK cells cooperate to produce synergistic killing of both AMJ13 and MCF-7 breast cancer cells (Figures 2E, 3E), while allowing for significant dose reductions of each component.

### AO/PI live-dead assay reveals apoptotic cell death

**FIGURE 4:**
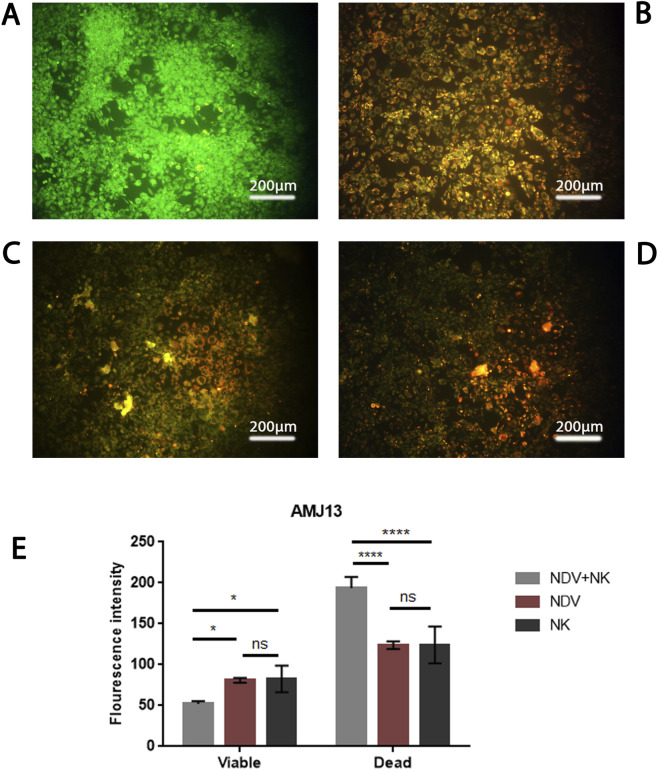
AO/PI dual staining demonstrates apoptotic killing of AMJ13 cells by NDV-NK co-treatment. **(A)** Untreated control shows predominantly green acridine orange (AO) fluorescence, indicating intact membrane viability. **(B,C)** NDV alone (MOI 20) or NK cells alone (2000 cells/well) produces a mixed green/orange pattern with scattered PI-positive cells, consistent with partial induction of apoptosis and limited late-stage cell death. **(D)** Combined NDV + NK treatment (MOI 20 + 2000 NK cells/well) markedly increases orange/red propidium iodide (PI) uptake and condensed/fragmented nuclear morphology, consistent with late apoptosis/secondary necrosis and loss of membrane integrity. **(E)** Quantification of fluorescence intensity (AO = viable; PI = dead/late apoptotic) confirms a significant reduction in AO signal and an ∼ two-fold increase in PI intensity in the NDV + NK group relative to either monotherapy (*p < 0.05; ****p < 0.0001; one-way ANOVA with Tukey’s multiple comparisons; mean ± SD; n = 3 independent fields). Scale bar = 200 µm.

**FIGURE 5:**
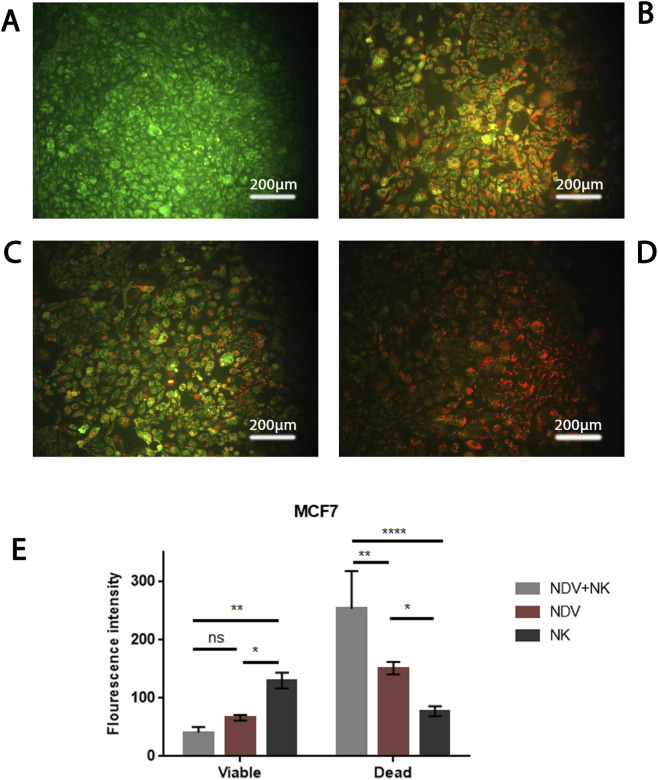
NDV-NK co-treatment enhances apoptotic cell death in MCF-7 breast carcinoma cells. **(A)** Untreated control cells display predominantly green acridine orange (AO) fluorescence, consistent with intact membrane integrity and viable nuclei. **(B,C)** Treatment with NDV alone (MOI 20) or NK cells alone (2000 cells/well) induces moderate apoptotic changes, characterized by mixed green/orange fluorescence and scattered propidium iodide (PI)-positive nuclei, indicating partial late-stage apoptosis. **(D)** Combined NDV + NK treatment (MOI 20 + 2000 NK cells/well) results in widespread red/orange PI uptake and condensed nuclear morphology, consistent with extensive late apoptosis and secondary necrosis. **(E)** Quantitative fluorescence intensity analysis demonstrates a significant reduction in AO-positive viable signal and a greater than two-fold increase in PI-positive fluorescence in the NDV + NK group compared to either monotherapy (**p < 0.01; ****p < 0.0001; one-way ANOVA with Tukey’s multiple comparisons; mean ± SD; n = 3 independent microscopic fields per condition). Scale bar = 200 µm.

To further characterize the mode of cell death, AO/PI dual staining was performed. In AMJ13 controls, nuclei fluoresced uniformly green, indicating intact membranes and viable cells (Figure 4A). NDV or NK monotherapy produced mixed green-orange staining, consistent with partial apoptosis and limited membrane damage (Figures 4B,C). In contrast, NDV-NK co-treatment induced widespread red/orange PI uptake (Figure 4D), reflecting advanced apoptosis and loss of membrane integrity. Quantitative fluorescence confirmed these findings: the combination significantly decreased viable AO-positive signal while doubling PI-positive intensity compared with single treatments (*p < 0.05; ****p < 0.0001) (Figure 4E). In MCF-7 cultures, a similar pattern emerged (Figure 5A). NDV or NK alone induced modest apoptotic responses with partial PI positivity (Figures 5B,C), whereas NDV-NK co-treatment markedly elevated red/orange nuclear staining, indicating extensive late apoptosis and/or secondary necrosis (Figure 5D). Quantitative analysis demonstrated >2-fold increase in PI-positive signal and significant reduction in viability relative to monotherapies (**p < 0.01; ****p < 0.0001) (Figure 5E). Thus, AO/PI assays not only confirm the WST and CompuSyn findings but also demonstrate that NDV-NK synergy enhances tumor killing through apoptosis-linked mechanisms.

### Morphological evidence of NK-Tumor interactions (NK adhesion assay)

**FIGURE 6:**
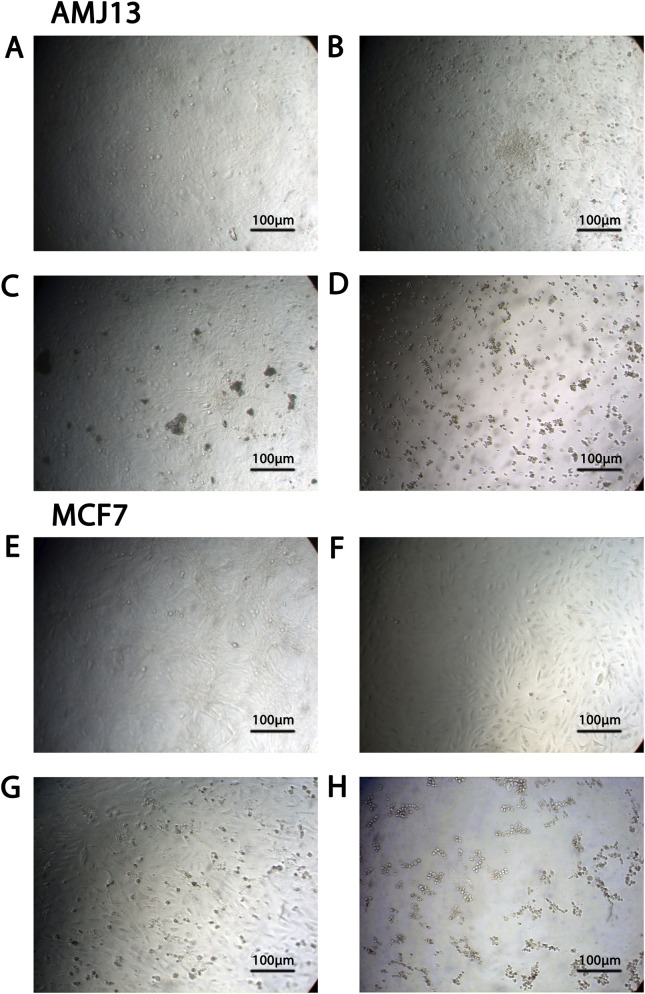
Light microscopy reveals enhanced NK-tumor interaction under NDV priming. Representative bright-field micrographs of AMJ13 **(A–D)** and MCF-7 **(E–H)** cells under the indicated treatment conditions. Images were acquired using a Micros Austria inverted light microscope equipped with a digital camera. **(A,E)** Untreated control cells display intact epithelial monolayers with preserved cell-cell junctions and normal morphology. **(B,F)** NDV alone (MOI 20) induces localized cytopathic effects characterized by membrane irregularities, cell rounding, and partial detachment in the absence of effector cells. **(C,G)** NK cells alone (2000 cells/well) result in limited tumor cell detachment, with small rounded lymphoid cells consistent with NK morphology visible within the tumor monolayer. **(D,H)** Combined NDV (MOI 20) + NK cells (2000 cells/well) produce pronounced cell shrinkage, widespread detachment, and marked architectural disruption. Multiple small round effector cells consistent with NK morphology are observed in close proximity to, and attached to, tumor cell surfaces, confirming effector cell presence and supporting infection-enhanced effector target interaction. Scale bar = 100 µm.

Light microscopy revealed direct morphological evidence of NK cell-tumor engagement following NDV priming. Untreated AMJ13 and MCF-7 cultures displayed intact monolayers with typical epithelial morphology and well-defined cell borders (Figures 6A,E). NDV alone induced localized membrane alterations and cell rounding (Figures 6B,F), while NK cells alone produced scattered clusters of detached or rounded tumor cells (Figures 6C,G). The most pronounced changes were observed under combined NDV–NK treatment (2000 NK cells + MOI 20 NDV). Both AMJ13 and MCF-7 cultures exhibited widespread cell detachment, membrane disruption, and shrinkage of tumor cells (Figures 6D,H). Rounded lymphoid cells morphologically consistent with NK cells were readily identifiable within co-culture conditions and were absent in NDV-only controls, confirming effector cell presence and distinguishing immune-mediated engagement from viral cytopathic effects alone. Numerous NK cells were seen adherent to tumor cell surfaces, indicating active immune–tumor interactions. These features are consistent with NDV enhancing tumor susceptibility to NK recognition and lysis, in line with the synergistic cytotoxicity observed in WST and AO/PI assays.

### Immunofluorescent detection of CD56 confirms NK engagement with NDV-Infected tumor cells

**FIGURE 7:**
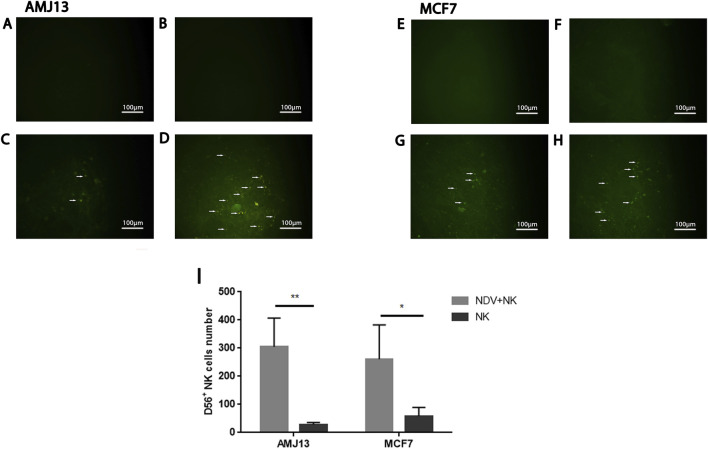
CD56 immunofluorescence demonstrates enhanced NK attachment to NDV-infected tumor cells. Representative immunofluorescence micrographs of AMJ13 **(A–D)** and MCF-7 **(E–H)** cells stained for CD56 (green) under the indicated treatment conditions. **(A,E)** Untreated tumor cells show negligible CD56 staining, confirming absence of NK cells. **(B,F)** NDV alone (MOI 20) does not produce CD56 signal, confirming antibody specificity. **(C,G)** NK cells alone (2000 cells/well) display modest CD56-positive signal following washing, indicating limited NK retention on tumor monolayers. **(D,H)** Combined NDV (MOI 20) + NK cells (2000 cells/well) markedly increases CD56 fluorescence, with visible clusters of CD56^+^ NK cells remaining attached to tumor cell surfaces after washing. Scale bar = 100 μm. **(I)** Quantitative analysis of retained CD56^+^ NK cells per microscopic field demonstrates significantly increased wash-resistant NK attachment following NDV priming. AMJ13 cultures exhibited greater than 10-fold enrichment of retained NK cells compared with NK-only treatment (**p < 0.01), while MCF-7 cultures showed approximately two-fold increased NK retention (*p < 0.05). Data represent mean ± SD derived from three independent microscopic fields per condition from separate wells. Statistical significance was determined by one-way ANOVA followed by Tukey’s multiple comparisons test.

Immunofluorescence staining for CD56 was performed to confirm NK cell presence and evaluate spatial effector-target interactions within adherent tumor monolayers. In untreated AMJ13 and MCF-7 cultures (Figures 7A,E), minimal CD56 signal was detected, consistent with the absence of NK cells. NDV treatment alone (MOI 20) did not generate CD56 staining in the absence of effector cells (Figures 7B,F), confirming staining specificity for NK lineage cells. Cultures exposed to NK cells alone (2000 cells/well) demonstrated modest CD56 positivity, indicating limited NK retention following washing (Figures 7C,G). In contrast, NDV-infected tumor cultures co-treated with NK cells displayed a pronounced increase in CD56 fluorescence intensity and visible clustering of CD56^+^ cells in close contact with tumor surfaces (Figures 7D,H). Importantly, following gentle washing to remove non-adherent cells prior to fixation, significantly higher numbers of CD56^+^ NK cells remained associated with NDV-infected monolayers compared with NK-only conditions, indicating enhanced wash-resistant NK-tumor attachment under NDV priming. Quantitative analysis of CD56^+^ NK cells per microscopic field (derived from three independent microscopic fields per condition from separate wells) confirmed this observation (Figure 7I). In AMJ13 cultures, NDV-NK co-treatment resulted in greater than ten-fold enrichment of retained NK cells relative to NK-only controls (**p < 0.01). Similarly, in MCF-7 cultures, NDV priming produced approximately two-fold higher NK cell retention compared with NK-only treatment (*p < 0.05).

While CD56 staining identifies NK lineage cells and their spatial localization rather than adhesion molecule engagement or activation state, the increased wash-resistant NK attachment observed in NDV-infected cultures correlates with the enhanced cytotoxicity detected in WST and AO/PI assays. Together, these findings support a cooperative interaction in which NDV infection promotes a tumor microenvironment permissive to stable NK-tumor cell contact and amplified cytotoxic activity.

### NDV infection confirmed by HN immunofluorescence in breast cancer cells

**FIGURE 8:**
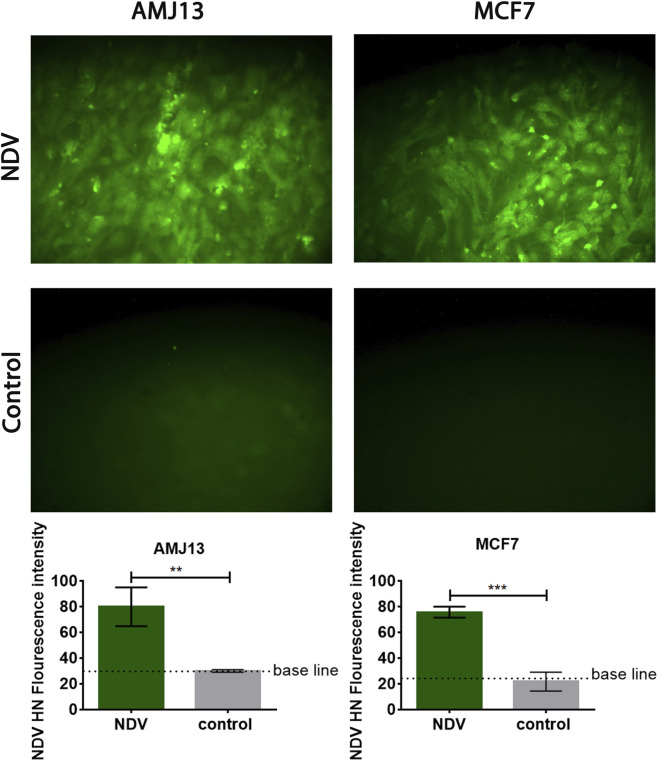
Immunofluorescent detection of NDV hemagglutinin-neuraminidase (HN) confirms productive infection in AMJ13 and MCF-7 breast cancer cells. Representative fluorescence micrographs showing HN expression (green) in AMJ13 and MCF-7 cells following NDV infection (MOI 20) or untreated control conditions. Strong HN signal is observed exclusively in NDV-infected cultures, whereas control cells exhibit minimal background fluorescence. All images were acquired under identical exposure and gain settings. Image quantitative analysis of mean HN fluorescence intensity per microscopic field confirms significant induction of viral protein expression in infected cells. Data represent mean ± SD derived from three independent microscopic fields per condition from separate wells. Statistical analysis was performed using unpaired Student’s t-test (**p < 0.01; ***p < 0.001). Scale bar = 100 μm.

To verify productive NDV infection in both tumor models, immunofluorescent staining for the viral hemagglutinin-neuraminidase (HN) protein was performed. Strong cytoplasmic HN fluorescence was observed in NDV-infected AMJ13 and MCF-7 cells, whereas untreated controls showed only minimal background signal (Figure 8). Quantitative analysis demonstrated a significant increase in HN fluorescence intensity in infected cultures compared to controls in both AMJ13 (**p < 0.01) and MCF-7 (***p < 0.001) cells. These findings confirm efficient viral protein expression under the experimental conditions used for NK co-culture assays.

Importantly, the confirmation of infection status provides mechanistic context for the enhanced NK clustering observed in subsequent immunofluorescence and SEM analyses, supporting the interpretation that NK engagement occurs preferentially in NDV-infected tumor regions rather than as a consequence of nonspecific cytopathic disruption.

### Ultrastructural evidence of enhanced NK engagement with NDV primed breast cancer cells

**FIGURE 9:**
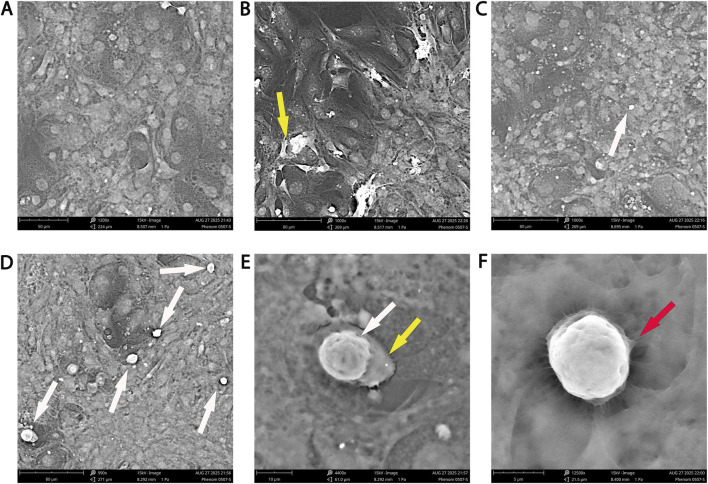
Scanning electron microscopy of AMJ13 breast cancer cells under different treatment conditions. **(A)** Untreated control showing intact monolayers with smooth cell surfaces. **(B)** NDV infection alone induces membrane blebbing and collapse and cells detachment (yellow arrow). **(C)** NK cells alone show rare adherence to uninfected tumor cells, with few NK cells visible on the surface (white arrow). **(D)** NDV + NK co-treatment results in multiple NK cells adhering simultaneously (white arrows). **(E)** Higher magnification view of NDV-infected tumor cell with an NK cell tightly attached (white arrows); tumor surface shows localized disruption (yellow arrows). **(F)** High-magnification NK cell displaying close apposition and synapse formation with the tumor surface (red arrow). Scale bars: 50–5 µm as indicated in each image.

Scanning electron microscopy provided ultrastructural evidence of NDV-NK synergistic interaction in AMJ13 breast cancer cells (Figure 9). Untreated controls (Figure 9A) displayed smooth tumor cell surfaces with preserved integrity. NDV infection alone (Figure 9B) induced membrane blebbing and surface collapse, consistent with direct viral cytopathic effects. NK cells added to uninfected AMJ13 cultures adhered only sporadically, with single effector cells visible on otherwise intact tumor surfaces (Figure 9C). On the other hand, NDV-infected AMJ13 cultures co-incubated with NK cells showed more NK cell attachment, with multiple effectors clustering across tumor surfaces (Figure 9D). High-magnification views revealed NK cells forming tight immune synapses, characterized by intimate membrane apposition and localized tumor cell disruption (Figures 9E,F). Target cells under NK contact zones exhibited roughened surfaces, pore-like lesions, and cytoplasmic collapse. Together, these ultrastructural findings confirm that NDV primes tumor cells for enhanced NK recognition and facilitates robust NK-mediated cytolysis.

**FIGURE 10:**
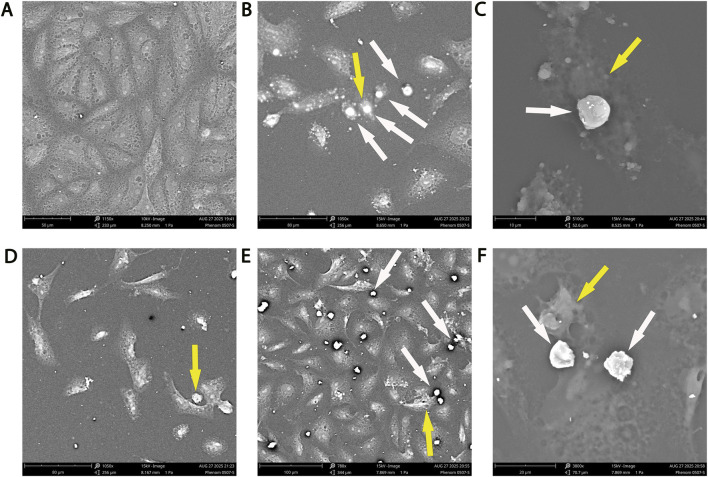
Scanning electron microscopy of MCF-7 breast cancer cells demonstrates enhanced NK-tumor interactions with NDV priming. **(A)** Untreated MCF-7 cells showing intact monolayer morphology. **(B)** NK cells alone adhere sporadically to tumor cells (white arrows) and attach to dying tumor cells (yellow arrow) which may indicate NK cells direct killing effect against breast cancer cells. **(C)** High-magnification view of NK cell (white arrow) tightly attached to non-infected tumor cell surface which showing blebs of programed cells death. **(D)** NDV infection alone induces tumor cell rounding and apoptotic-like morphology (yellow arrow). **(E)** NDV + NK co-treatment results in extensive NK adherence (white arrows) and preferential attachment to apoptotic NDV-infected tumor cells (yellow arrow). **(F)** High-magnification SEM image showing NK cells (white arrows) clustered on an NDV-infected tumor cell with presence of tumor cell death (yellow arrow). Scale bars: 50–10 µm as indicated.

SEM analysis of MCF-7 breast carcinoma cells supported the enhanced NK–tumor interactions observed in AMJ13 (Figure 10). Untreated controls (Figure 10A) exhibited confluent monolayers with intact, smooth surfaces. In the presence of NK cells alone, a small number of NKs were adherent to tumor cells (Figure 10B), with some preferentially attaching to rounded, degenerating tumor cells. Higher magnification confirmed NK cells in close apposition to tumor cell surfaces, forming effector-target contacts (Figure 10C). NDV infection alone induced morphological changes in MCF-7, including cell rounding, detachment, and membrane roughening indicative of cytopathic damage (Figure 10D). The most striking changes occurred with combined NDV + NK treatment (Figures 10E,F), where numerous NK cells were seen clustering on tumor monolayers and attaching preferentially to apoptotic or degenerating NDV-infected tumor cells. At higher magnification, NK cells were observed forming tight immune synapses, with tumor membranes collapsing under effector contact zones. These ultrastructural features reinforce the concept that NDV infection primes tumor cells for NK recognition and augments NK-mediated cytolysis.

## Discussion

The present study extends prior knowledge of NDV immunomodulation by providing quantitative synergy mapping, dose-sparing analysis, and ultrastructural evidence of NK engagement in two breast cancer models. In both breast cancer models (AMJ13, MCF-7), we show that AMHA1 attenuated NDV and naturally migrated isolated NK cells synergize to kill tumor cells far more effectively than either alone. Synergy was clear as CI values below 1 for most dose pairs with meaningful dose-reduction indices (DRI) for both agents, indicating true therapeutic gain rather than additive effects. All the experiments conducted including viability assay, AO/PI apoptosis, NK CD56 IF cell-cancer cell adhesion assay, light microscopy, and SEM examination reached on the same conclusion: NDV primes tumor cells for NK recognition, creating more stable NK-tumor contacts, and accelerates cytolytic destruction. These data position NDV-NK immunovirotherapy as a plausible translational strategy for breast cancer therapy. The Chou–Talalay equation (Chou, 2010) used for NDV-NK combinations to study their interaction (CI) and dose-sparing (DRI). Our DRIs suggest that clinically relevant tumor inhibition could be achieved at most doses especially the lower doses of both NDV and NK, which is promising for cell therapy and dosing limits for oncolytic viruses. Methodologically, our dose–effect, Fa–CI, isobologram, and DRI outputs follow best practice for preclinical combination development and provide a transparent bridge to first-in-human design.

Microbial platforms capable of modifying extracellular matrix architecture or locally concentrating cytotoxic agents have shown the capacity to increase CD8^+^ T cell infiltration and improve therapeutic response independent of tumor epitope status. These studies underscore a growing paradigm in which biologically targeted delivery systems function not only as direct cytotoxic agents but also as immune modulators within the tumor microenvironment (Thomas et al., 2022; Siddiqui et al., 2023). Within this conceptual framework, attenuated NDV represents a viro-immunotherapeutic analogue capable of tumor-selective infection, induction of immunogenic cell death, and enhancement of innate immune effector engagement. Our findings extend this paradigm by demonstrating that NDV infection promotes stable NK-tumor cell interaction and synergistic cytotoxicity, supporting the integration of oncolytic virotherapy with adoptive or endogenous NK-based immunotherapy.

Productive viral infection was independently confirmed by immunofluorescent detection of the NDV hemagglutinin-neuraminidase (HN) protein in both AMJ13 and MCF-7 cells. HN expression was robust in infected cultures and absent in controls, confirming active viral replication under the experimental conditions. Importantly, the spatial correlation between HN-positive tumor regions and enhanced NK accumulation supports the interpretation that NDV-mediated modulation of tumor cell surface properties contributes to improved effector-target engagement. While we did not directly assess NK activating receptors (NCR) such as NKp30, NKp44, or NKp46, the presence of viral surface glycoproteins provides a biologically plausible mechanism for enhanced NK recognition, consistent with prior reports describing NDV-NCR interactions.

NDV is an immunogenic oncolytic virus, act by inserting its HN and F proteins at the tumor cells surface during its replication cycle leading for tumor cells antigenic modification (Alshammari et al., 2024). lyses tumor cells while activating type-I IFN pathways and danger signaling that recruit innate lymphocytes, including NK cells (Biswas et al., 2012).

Importantly, light microscopy independently confirmed the physical presence of NK cells within tumor monolayers, supporting interpretation of enhanced effector–target interaction rather than viral cytopathic effects alone. At the tumor-cell surface, NDV can increase expression of ligands for activating NK receptors, thereby enhancing immune-synapse formation and contact-dependent killing, which is consistent with our CD56 adherence counts and SEM images showing multiple NKs opposed to infected targets. Previous work has demonstrated NDV-induced up-modulation of NK-activating ligands on infected tumor cells and NK activation after NDV exposure (Jarahian et al., 2009), supporting our observation that NDV infection sensitized NK cells to attack tumor cells leading for enhanced killing. The isolated effectors were CD3^−^CD56^+^ with CD16 (cytotoxic subset) and CD57 (maturation/activation) expression is an immunophenotype associated with potent cytolysis (Kared et al., 2016). Immunophenotypic characterization confirmed that the effector population consisted predominantly of CD3^−^CD56^+^ NK cells with detectable CD16 and CD57 expression, supporting enrichment of cytotoxic and partially mature NK subsets. Although fluorescence microscopy does not permit quantitative resolution of CD56^bright^ versus CD56^dim^ subsets, the presence of CD16^+^ cells suggests inclusion of cytotoxic NK populations commonly associated with target cell lysis. CD57 expression further indicates a degree of functional maturation within the expanded NK compartment. Importantly, our interpretation of NK phenotype is based on marker co-detection rather than fluorescence intensity stratification, and we therefore avoid inferring subset distribution beyond what can be supported by imaging data. Although CD16 and CD57 were detected within the NK population, the present study did not quantify the exact proportion of effector CD16^+^ NK cells by multiparameter flow cytometry. Therefore, we refrain from reporting subset percentages. Functional cytotoxic competence was instead supported by WST-based killing assays, Chou-Talalay synergy modeling, AO/PI apoptosis validation, and ultrastructural effector-target engagement. Future studies incorporating flow cytometric co-expression analysis of CD3, CD56, CD16, CD57, perforin, and granzyme B will allow more precise quantification of NK subset distribution. CD16A serves as a highly effective activating receptor on natural killer (NK) cells (Wu et al., 2020). Together with increased CD56^+^ cell adherence under NDV priming, these features provide a cellular basis for the functional synergy we reported. NK-cell therapies translated from concept to early clinical application, with multiple modalities (autologous, allogeneic, cytokine-primed, iPSC-derived, and engineered NKs) showing encouraging safety and signals of efficacy (Shimasaki et al., 2020; Page et al., 2024). Moreover, oncolytic viruses have advanced clinically (e.g., T-VEC approval; expanding OV pipelines), with increasing importance on immunostimulatory mechanisms and rational combinations (Al-Shammari and Piccaluga, 2025). Recent review (Franks et al., 2024) highlight the opportunity to pair OVs with adoptive cell therapies, including NKs, to convert cold tumors and augment cytotoxicity, which is what we precisely found by our current work.

Our results support a rational sequencing hypothesis: virus first, NK second. *In vitro*, NDV priming increased NK adherence and killing; clinically, this could translate to intratumoral NDV administration followed by timed NK infusion into the same lesion or vascular compartment. This study is *in vitro*, on 2D monolayers, and uses sub-stoichiometric NK:tumor ratios; and it does not capture stromal barriers and antiviral immunity. MOI values do not translate directly to patient dosing. Finally, While NDV generally enhances NK recognition by increasing NKG2D-ligand expression, this effect cannot be assumed universally. Other viruses like reovirus can cause the opposite effect, depending on the tumor type and host factors (Khaleafi et al., 2023). Therefore, before moving NDV-NK therapy into clinical trials, we need to verify in patient-derived tissues and *in vivo* that NDV infection indeed upregulates (not downregulates) these ligands, to confirm that the synergy we see *in vitro* will hold true in real human tumors.

We acknowledge that multiparametric flow cytometry would provide higher-resolution characterization of NK subsets, including quantitative discrimination of CD56^bright^ versus CD56^dim^ populations, assessment of CD16 co-expression frequencies, and evaluation of activation and degranulation markers such as CD107a, perforin, and granzyme B. Due to limited access to flow cytometry instrumentation during the study period, NK phenotyping was performed using multi-marker immunofluorescence microscopy (CD3, CD56, CD16, CD57), which enabled spatial confirmation of lineage identity and maturation status but did not permit precise subset frequency quantification. Similarly, while CD56 immunofluorescence was used as a lineage and localization marker, it was not intended as a stand-alone surrogate for adhesive strength or functional activation. To strengthen infection specificity, we incorporated immunofluorescent detection of NDV hemagglutinin-neuraminidase (HN), confirming viral presence in tumor cells and supporting interpretation of enhanced NK engagement under NDV priming. Future studies will incorporate multiparameter flow cytometry to quantify NK subset distribution, degranulation responses, and receptor expression, as well as molecular analysis of NKG2D ligand regulation using Western blotting, ELISA, and immunocytochemistry to further dissect virus-induced tumor susceptibility mechanisms.

**FIGURE 11:**
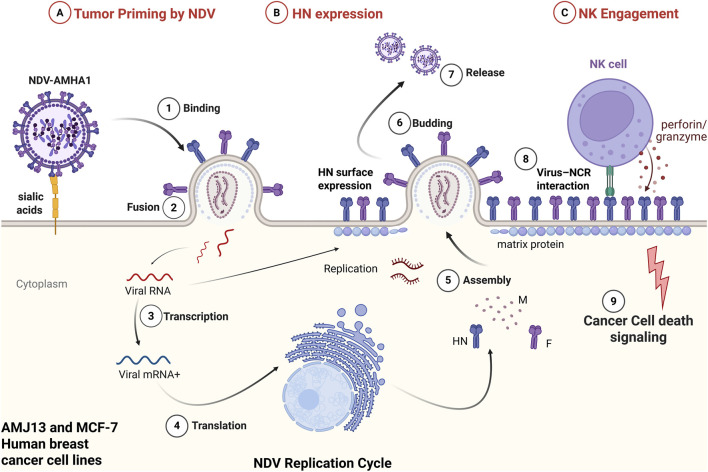
NDV replication and NK cell engagement model in AMJ13 and MCF-7 breast cancer cells. **(A)** Tumor priming by NDV-AMHA1 begins with viral binding to sialic acid residues on the tumor cell surface (1), followed by membrane fusion (2) and release of viral RNA into the cytoplasm. Viral transcription (3) and translation (4) drive synthesis of structural proteins, including hemagglutinin-neuraminidase (HN), fusion (F), and matrix (M) proteins. Viral assembly (5), budding (6), and release (7) complete the replication cycle. **(B)** Productive infection results in surface expression of viral HN glycoprotein on tumor cells, altering membrane composition and immunogenicity. **(C)** NK engagement: HN-expressing tumor cells establish virus-natural cytotoxicity receptor (NCR) interactions (8), facilitating stable NK-tumor contact. NK cells subsequently release perforin and granzymes, triggering apoptotic signaling cascades and tumor cell death (9). This model summarizes the mechanistic basis for the enhanced cytotoxicity observed experimentally in NDV-NK combination treatments, integrating viral replication, tumor surface remodeling, NK recognition, and immune-mediated tumor cell killing. Created in BioRender. Al-Shammari, A. (2026) https://BioRender.com/gytwkyg.

Conclusion. By integrating quantitative synergy metrics with cellular and ultrastructural evidence, we demonstrate that NDV reshapes tumor cells into highly permissive targets for NK cytolysis and that NDV-NK cooperativity is both mechanistically coherent and dose-efficient. These properties are exactly what is required for a clinically testable, modular regimen that can be layered onto the current NK therapy and oncolytic virus landscapes. Based on our experimental findings, we propose a mechanistic model whereby NDV infection primes tumor cells for enhanced NK recognition and cytotoxic engagement (Figure 11).

## Data Availability

The original contributions presented in the study are included in the article/supplementary material, further inquiries can be directed to the corresponding author.
